# The educational value of inpatient psychiatric training in undergraduate medical education

**DOI:** 10.5116/ijme.5869.00e7

**Published:** 2017-02-18

**Authors:** Noah Capurso, Kirsten Wilkins

**Affiliations:** 1 Department of Psychiatry, School of Medicine, The Yale University, New Haven, CT, USA

**Keywords:** educational value, inpatient, psychiatric training, undergraduate medical education

## Introduction

The core psychiatry clerkship, typically held during the third year of medical school, serves as the primary opportunity for medical students to gain exposure to the clinical practice of psychiatry. Multiple studies have evaluated the effect that the clerkship has on students, and a clear body of evidence indicates that the clerkship influences students’ attitudes towards the field of psychiatry.^1^ While studies have typically found that clerkships result in a more favorable view of psychiatry as a practice^2^^-^^4^, the specific components that students find valuable have not been examined in nearly as much detail.

### Problem

The structure and composition of clerkships in psychiatry and other disciplines have come under scrutiny in recent years, as medical schools undergo curriculum restructuring.^5^^-^^6^ An increasingly evolving field of medicine dictates that medical schools must educate students in new and innovative ways.^7^ Educators have challenged the longstanding tradition of a 12-month clerkship year spent in hospitals with a growing emphasis on training students where the majority of healthcare is provided: the ambulatory care setting.^8^^,^^9^ The effect of these trends on student training in psychiatry is not known.

Our home institution is among the many schools that have recently implemented large-scale curriculum redesigns, including significant changes to the structure of the clinical clerkships. Given the growing emphasis on medical training in the ambulatory setting, preliminary discussions regarding psychiatry clerkship restructuring raised questions about the necessity of inpatient psychiatric training for all clerkship students. Some faculty questioned how valuable such training is for the general, “undifferentiated” medical student. In light of these questions and the environment of curriculum change, we sought to investigate the educational value of the inpatient portion of the psychiatry clerkship, as perceived by students. This paper presents the results of our investigation and a compelling argument for the continuation of inpatient psychiatric training in undergraduate medical education.

### Lessons learned

Feedback from medical students indicated they found the inpatient psychiatry rotation largely enjoyable and educational. Students reported a high rate of exposure to core psychiatric clinical experiences identified by the authors as valuable in their development as physicians (e.g., exposure to patients with suicidal ideation and agitation, learning to tolerate challenging patient affect, considering psychosocial contributors to illness and treatment, etc.). Interestingly, responses from students who indicated a particular interest in psychiatry did not differ from students who did not indicate an interest in pursuing psychiatry. Nearly all students felt that inpatient psychiatry should be a required portion of the third year clerkship.

One frequently recognized experience that students found valuable was the ability to round in interdisciplinary teams. Students commented that the degree of interdisciplinary interactions in daily work and on rounds was greater than that experienced on other clerkships. This is especially valuable considering the increasing interdisciplinary nature of modern medical care as highlighted by the Liaison Committee on Medical Education’s 2016 Accreditation Standards Element 7.9 that requires students to function collaboratively on teams that include professionals from other disciplines.^10^

Students also reported value in seeing the “continuum of care” for psychiatric patients, from outpatient evaluations to the inpatient unit. Having an experience in inpatient psychiatry helped them accurately educate patients and address their concerns about hospitalization. Students reported that working with psychiatric inpatients for a significant amount of time served to destigmatize patients with the severe mental illness. Finally, students reported that they were able to get to know patients in a more detailed and intimate manner on an inpatient unit than in the psychiatric emergency department or on the consult service, where they might see a given patient on only a handful of occasions.

## Conclusions

In light of these findings, it is our opinion that inpatient psychiatry is an essential component of undergraduate medical education. Regardless of their future career plans, medical students highly value the inpatient psychiatry rotation. Additionally, they report that many of the valuable educational components of this training cannot be experienced in other clinical rotations. We argue that care must be taken when restructuring medical school curricula so that valuable inpatient experiences are preserved and not sacrificed in the trend towards training in ambulatory settings.

### Acknowledgements

The authors would like to acknowledge Dr David A. Ross and Dr Robert M. Rohrbaugh for invaluable conversations and support.

### Conflicts of Interest

The authors declare that they have no conflict of interest.
